# Intelligent Monitoring System of Migratory Pests Based on Searchlight Trap and Machine Vision

**DOI:** 10.3389/fpls.2022.897739

**Published:** 2022-06-20

**Authors:** Guojia Sun, Shuhua Liu, Haolun Luo, Zelin Feng, Baojun Yang, Ju Luo, Jian Tang, Qing Yao, Jiajun Xu

**Affiliations:** ^1^School of Information Science and Technology, Zhejiang Sci-Tech University, Hangzhou, China; ^2^State Key Laboratory of Rice Biology, China National Rice Research Institute, Hangzhou, China

**Keywords:** searchlight trap, rice migratory pests, intelligent monitoring, deep learning, machine vision

## Abstract

Three species of rice migratory pests (*Cnaphalocrocis medinalis, Sogatella furcifera*, and *Nilaparvata lugens*) cause severe yield and economic losses to rice food every year. It is important that these pests are timely and accurately monitored for controlling them and ensuring food security. Insect radar is effective monitoring equipment for migratory pests flying at high altitude. But insect radar is costly and has not been widely used in fields. Searchlight trap is an economical device, which uses light to trap migratory pests at high altitude. But the trapped pests need to be manually identified and counted from a large number of non-target insects, which is inefficient and labor-intensive. In order to replace manual identification of migratory pests, we develop an intelligent monitoring system of migratory pests based on searchlight trap and machine vision. This system includes a searchlight trap based on machine vision, an automatic identification model of migratory pests, a Web client, and a cloud server. The searchlight trap attracts the high-altitude migratory insects through lights at night and kills them with the infrared heater. All trapped insects are dispersed through a multiple layers of insect conveyor belts and a revolving brush. The machine vision module collects the dispersed insect images and sends them to the cloud server through 4G network. The improved model YOLO-MPNet based on YOLOv4 and SENet channel attention mechanism is proposed to detect three species of migratory pests in the images. The results show that the model effectively improves the detection effect of three migratory pests. The precision is 94.14% for *C. medinalis*, 85.82% for *S. furcifera*, and 88.79% for *N. lugens*. The recall is 91.99% for *C. medinalis*, 82.47% for *S. furcifera*, and 85.00% for *N. lugens*. Compared with some state-of-the-art models (Faster R-CNN, YOLOv3, and YOLOv5), our model shows a low false detection and missing detection rates. The intelligent monitoring system can real-timely and automatically monitor three migratory pests instead of manually pest identification and count, which can reduce the technician workload. The trapped pest images and historical data can be visualized and traced, which provides reliable evidence for forecasting and controlling migratory pests.

## Introduction

According to the Food and Agriculture Organization of the United Nations, the annual potential loss of crop yield caused by pests is about 30% worldwide. Migratory pests are among the most harmful, as they can cause great disasters in a short period of time (Hu et al., 2020). Considering the long-distance migratory ability of migratory pests, dynamic monitoring of migratory pests’ population is crucial for timely and effective pest management. Dynamic monitoring of pest populations includes adult monitoring, field pest egg survey, and damage symptom investigation. Among them, timely monitoring of adult occurrence time and quantity is the basis of effective pest management (Jiang et al., 2021). At present, the adult monitoring equipment of migratory pests mainly includes insect radar and light trap. Insect radar mainly indirectly monitors pest species and quantity through calculating the insect flapping wing frequency, the body shape, and size of each insect in radar images (Feng, 2011; Zhang et al., 2017). In fact, the insect wing flapping frequency is related to insect instar and flight environment temperature. Insects with the same shape and size may be different insect species. Consequently, it is difficult to accurately identify the insect species which becomes a major obstacle to the widespread application of insect radar in fields for migratory pest forecasting (Feng, 2003). As an important tool for monitoring agricultural pests, light traps can be divided into two types (Yang et al., 2017). One is for trapping pests in fields, named ground light trap. The other is for trapping pests in high-altitude, named searchlight trap. Compared with the ground light trap, searchlight trap shows superiority in monitoring migratory pests, such as larger biomass, longer monitoring period, and more obvious fluctuation curve of pest quantity (Jiao et al., 2017; Shang, 2017; Qin, 2019). From 2014, searchlight traps (using metal halide lamps, bulb light source wavelength of 500–600 nm, and power of 1,000 W) have been used to monitor regional migratory pests and obtained good monitoring results (Jiang et al., 2016). However, the identification and count of pests trapped by the searchlight traps still needs to be carried out manually. This manual method requires high professional skills and spends much time, which causes low efficiency, high labor intensity and non-timely data application (Song et al., 2021; Yan et al., 2021).

With the development of machine vision, there has been some progress in pest detection and recognition studies based on images. Qiu et al. (2007) used the automatic threshold segmentation, feature extraction, and BP neural network classifier method to identify nine species of field pests. Based on the morphology and color features of pests, Han and He (2013) developed a support vector machine classifier to automatically identify six species of field pests. Zou (2013) adopted four different methods to extract shape features of rice planthoppers to improve the accuracy of pest identification. Yao et al. (2021a) proposed an automatic pest detection method based on improved CornerNet, which effectively improved the detection effect of rice planthoppers on light-trap insect images. Feng (2020) proposed YOLO-pest model to detect three species of *Cnaphalocrocis medinalis*, *Chilo suppressalis*, and *Sesamia inferens*, which reduced false detection and missing detection caused by insect adhesions in images. In order to improve the detection precision of light-trap insects, Yao et al. (2021b) proposed a bilinear attention network to identify similar light-tap pests. But there are no reports about intelligent searchlight traps based on machine vision and its pest identification methods. The challenges of pest identification from searchlight traps are (1) to timely disperse insects for collecting high-quality pest images, (2) to accurately identify the small size of pests, (3) to accurately distinguish those similar pests, and (4) to identify target pests from a large number of non-target insects.

To automatically identify and count rice migratory pests trapped by searchlight traps, we design an intelligent monitoring system of migratory pests based on searchlight trap and machine vision. The system can realize the automatic identification and count of three species of rice migratory pests (*C. medinalis, Sogatella furcifera*, and *Nilaparvata lugens*) attracted by searchlight trap.

## Materials and Methods

### Intelligent Monitoring System of Migratory Pests

**Figure 1:**
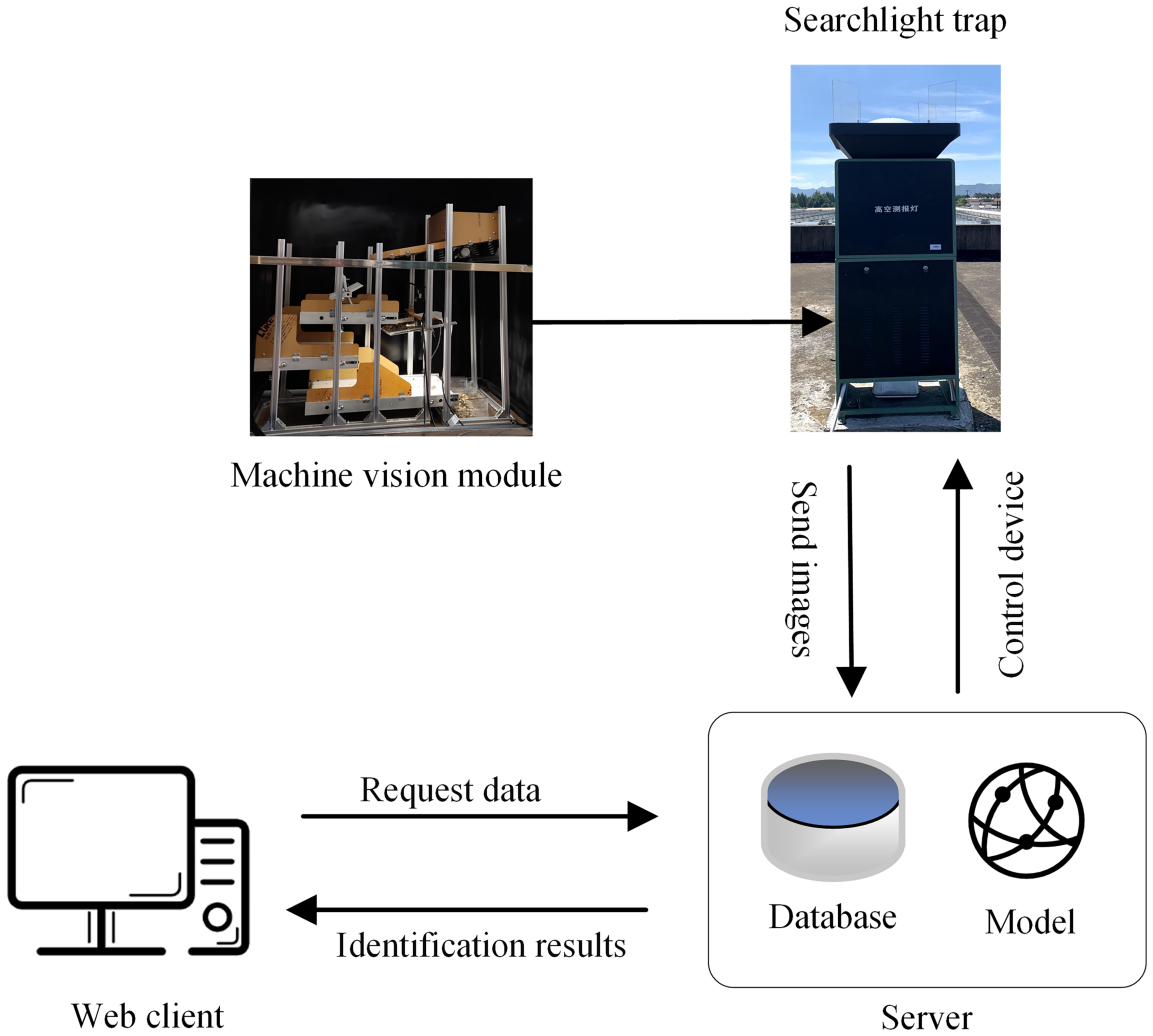
Intelligent monitoring system of rice migratory pests based on searchlight trap and machine vision.

The Intelligent monitoring system of migratory pests consists of an intelligent searchlight trap based on machine vision, an automatic identification model of migratory pests, a Web client, and a cloud server. The searchlight trap firstly attracts and kills insects, then the machine vision module disperses insects and captures images. After these images are uploaded to the server, the server runs the model to identify migratory pests in the images. Finally, the identification results of pests are presented to the Web client. Figure 1 shows the system construction.

The searchlight trap is mainly composed of a searchlight, an infrared heater module, and an insect collection box. When the equipment works at night, those flying insects within a high altitude of 500 m can be attracted. After the insects drop into the equipment from the top of the searchlight trap, they are killed and dried by the infrared heater module.

The machine vision module includes multilayer insect conveyor belts, Android PAD, industrial camera (MV-CE200-10GC), area light source, and network transfer module. Firstly, the dead insects fall into a vibrating slope controlled by a vibration motor for dispersing insects. Then, insects are dispersed onto the first layer of conveyor belts for further dispersion. Before falling onto the second layer of belt, the big insects are left and small insects are dispersed to the third layer of belt by a revolving brush for avoiding big insects covering small insects. When the big insects are transmitted onto the second layer, the camera takes photos of insects on the third layer of belt. The images are uploaded to the cloud server through the network transfer module on the Android PAD. The Android PAD is equipped with a special program to display images and photograph information (photograph time, image number, etc.) in real-time. The parameters can be manually set on the screen to control the photograph. Finally, all insects fall into the insect collection box at the bottom after they are photographed.

### The Image Dataset

The intelligent searchlight trap was installed in the paddy fields in Fuyang District, Zhejiang Province. 2,430 images with rice migratory pests were collected in 2021. The size of an image is 5,472 × 3,648 pixels.

The migratory pest images were divided into a training set and a testing set in the ratio of 9:1. We used the LabelImg tool to annotate three species of migratory pests (*C. medinalis, S. furcifera*, and *N. lugens*) in images. The classification information and coordinate information of the labeled region were saved in the corresponding XML file. The searchlight trap caught many non-target insects as well. Some of them are very similar to the target pests visually, which leads to false detection. These non-target insects are called interference pests in this paper. The information of dataset is shown in Table 1.

**Table 1:** The number of migratory pests on images.

Datasets	Image number	Pest number			
		*C. medinalis*	*S. furcifera*	*N. lugens*	Interference pests
Training sets	2,187	73,146	90,126	59,250	8,487
Test sets	243	6,993	9,006	5,850	822

### Image Preprocessing

#### Image Data Enhancement

**Figure 2:**
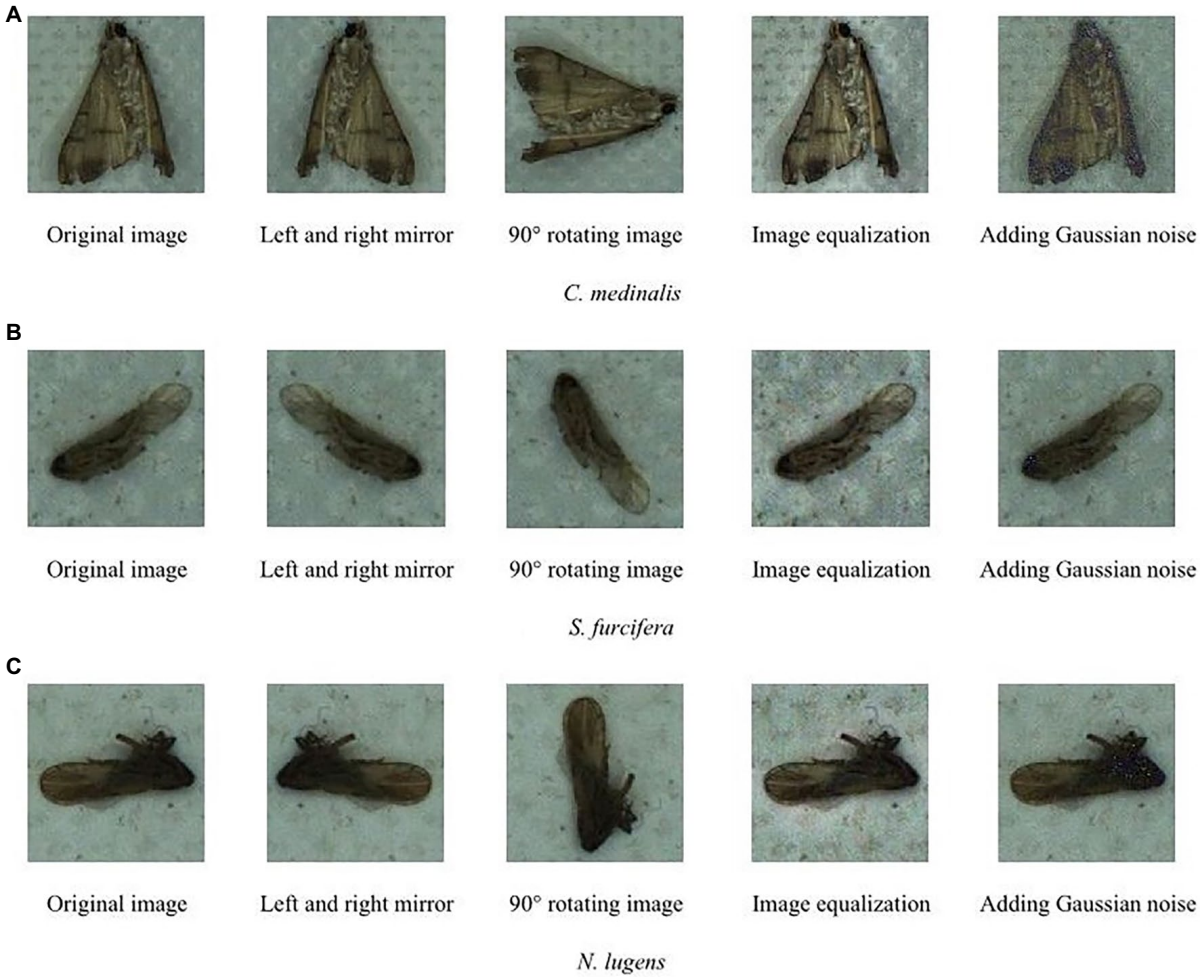
Data enhancement of target pest images **(A)** *C. medinalis*, **(B)** *S. furcifera*, and **(C)** *N. lugens*.

As we all know, the larger the dataset, the better the generalization performance for deep learning methods. To improve the robustness and generalization ability of the automatic identification model of migratory pests, we use some image processing methods to increase the number of images for training models. These methods include image left and right mirror, 90° rotating image, image equalization, and adding Gaussian noise (Lee, 1980). The algorithm functions of these methods are called from OpenCV library. Finally, the training sample number is increased by four times. The data enhancement results are shown in Figure 2.

#### Overlapping Sliding Window Method

Among three migratory pests, the size of two planthopper pests is about 3–5 mm, accounting for about 0.06% of the original image size. Due to the small area proportion of one pest in one image, the feature extraction network cannot extract effective features, which results in a concerning problem of missing detection of planthoppers. We adopt the overlapping sliding window processing method (OSW; Yao et al., 2021a) to improve the area proportion of each target pest in sub-images. The method can reduce missing detection and improve detection precision.

**Figure 3:**
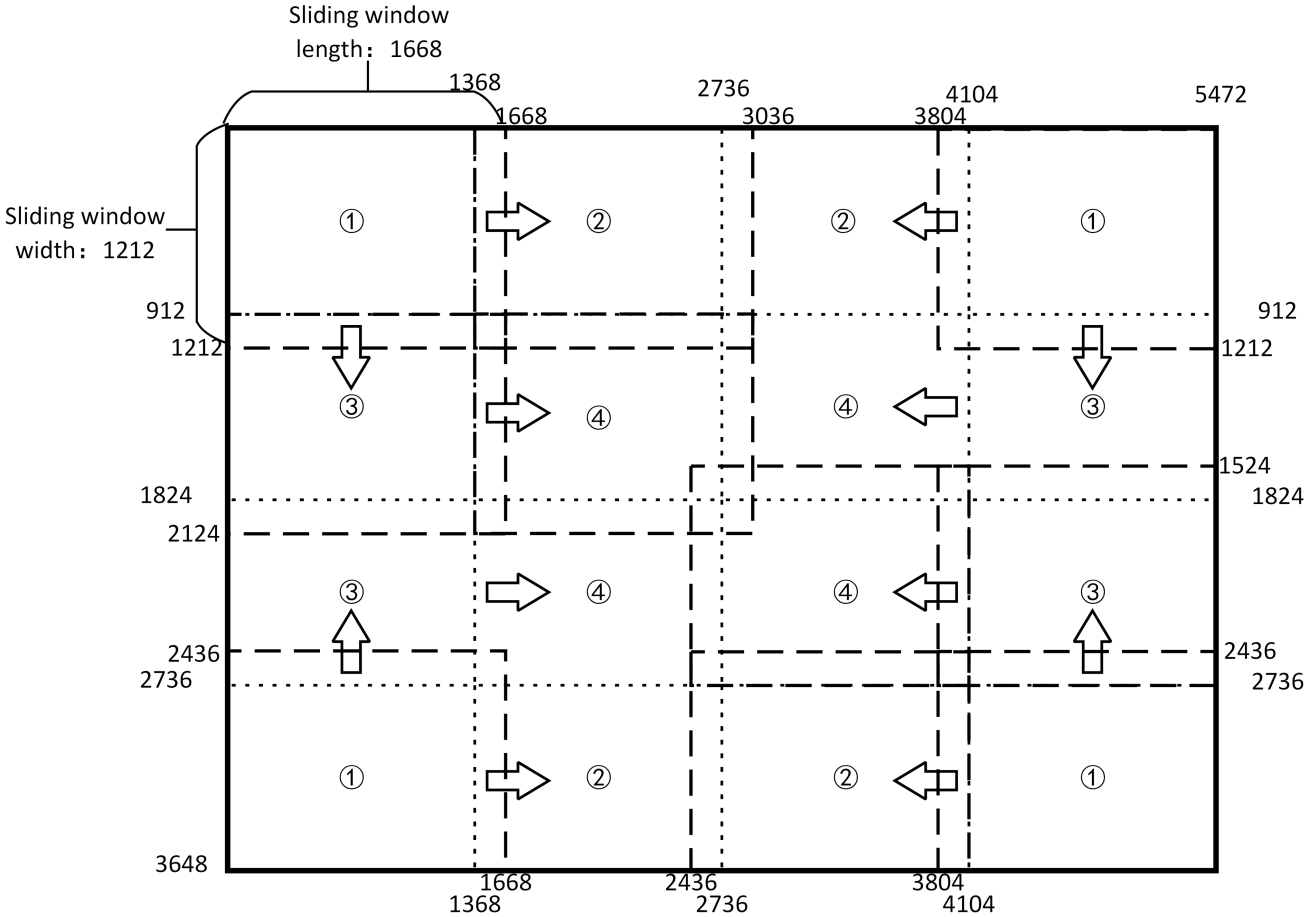
The diagram of overlapping sliding window method.

The original image is 5,472 × 3,648 pixels. We quarter the size of the original image and then add the length of the circumscribed rectangle of the largest pest (300 pixels) in the image to determine the size of the fixed window as 1,668 × 1,212 pixels. During detection, the sliding window slides towards the center from all sides. The order of movement is to move from the position (1) slides to (2) and (3) respectively, then slides from (3) to position (4). Figure 3 shows the implementation of overlapping sliding window processing method. It cuts out the image in the window to become a new subimage when sliding. The size of the new subimage is smaller than the original image, but the size of the pests in the subimage has not changed, so the area ratio of each target pest has increased. The change from small target into “large” target contributes to extract the features of the target more efficiently. In the example figure, the sliding window takes the image at position (1) and then the picture at position (2). An overlapping area of pest length is left between the two subimages, which ensures that each pest will be fully learned and detected at least once. In this way, the missing detection is reduced and the number of data sets can be increased without destroying the integrality of the insect body, which is beneficial for improving the detection precision of the model. If a target pest happens to appear on the boundary of the sliding window, part of the pest body appears in the sliding window and it may be detected by the detection box. This problem is subsequently solved by the target detection box suppression method.

### Detection Model of Rice Migratory Pests

#### Model Network Framework

Typical single-stage object detection models include the YOLO series (Redmon et al., 2016; Redmon and Farhadi, 2017, 2018), SSD (Liu et al., 2016), etc. The YOLO model is known for both speed and precision.

In our work, the YOLOv4 is used to detect three rice migratory pests from our intelligent searchlight trap based on machine vision. The YOLOv4 model consists of a feature extraction network CSPDarknet-53 and an up-sampling feature fusion module (Bochkovskiy et al., 2020). The activation function for DarknetConv2D of YOLOv4 is Mish and the convolution block is DarknetConv2D_BN_Mish. This design makes it not completely truncate at negative value, thereby ensuring information flow and avoiding the problem of saturation. YOLOv4 uses the CSPnet structure to enhance learning ability through repeated feature extraction. The SPP structure is added to the feature extraction network of YOLOv4, which can greatly increase the receptive field and isolate the most significant contextual features. These improvements enable YOLOv4 to achieve better detection results while consuming less computational resources. The migratory pest targets in this paper have the characteristics of large insect quantity, many insect species, small targets, and similar insects, which put forward higher requirements for the robustness and computational performance of the model. Accordingly, we chose YOLOv4 as the original detection model.

**Figure 4:**
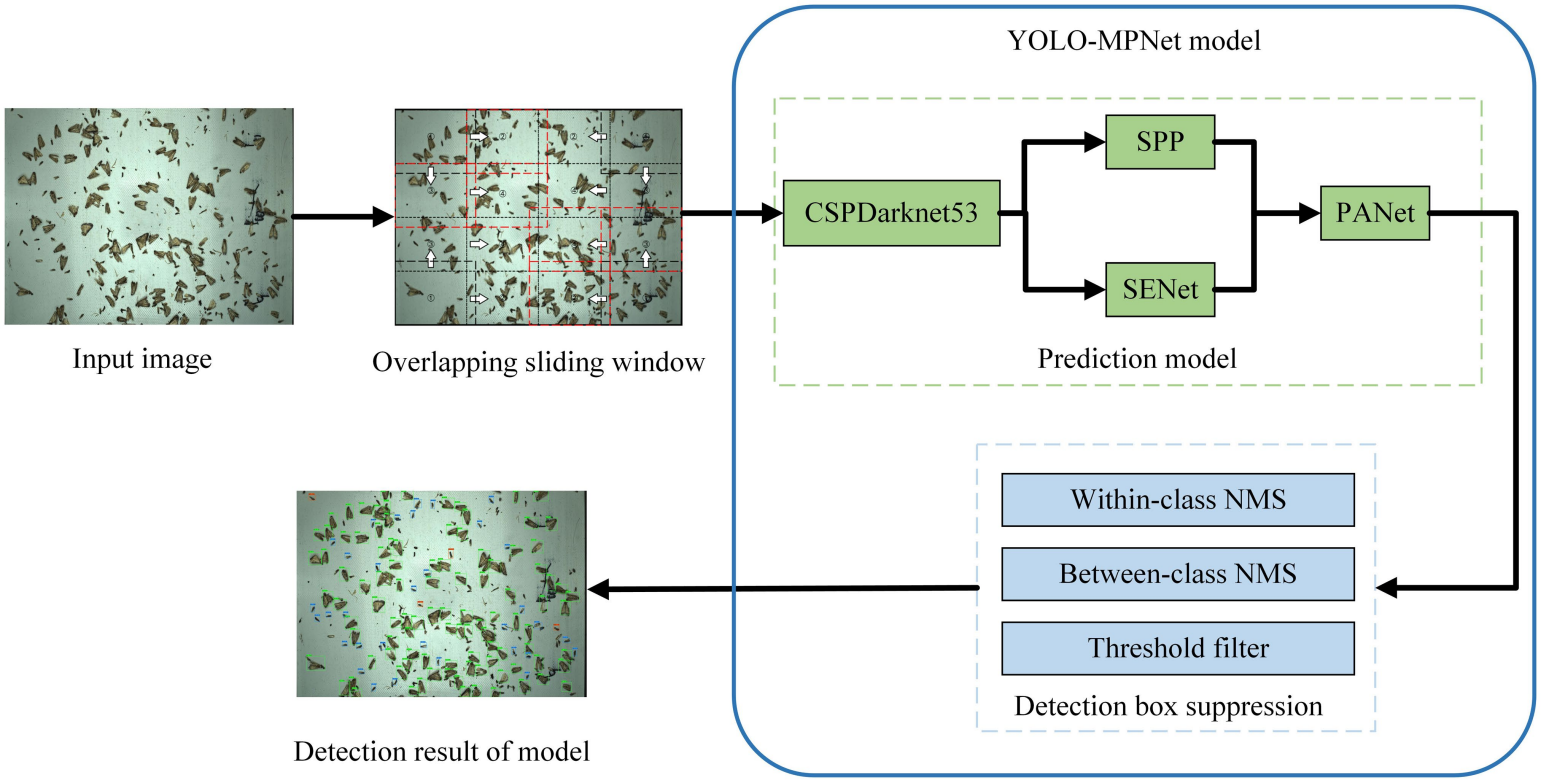
The network framework of YOLO-MPNet model.

Due to the complex background of migratory pests caused by lots of non-target insects trapped by searchlight trap, the original YOLOv4 model has two detection problems. One is the false detection of target pests and interference pests. The other is the missing detection of small target pests. Aiming at the two problems, we firstly use the overlapping sliding window method to increase the area proportion of the targets in one image. Secondly, the SENet channel attention mechanism is added to the YOLOv4 model to reduce the false detection of target pests. The improved model is named YOLO-MPNet and its network framework is shown in Figure 4.

By adding the SENet channel attention mechanism, we design one dependency model of each channel. This model improves the expression ability of the network and makes the network selectively learn some features. Besides, this new network structure can adaptively detect targets by slightly increasing model complexity and a small amount of computation. The specific steps are as follows: (1) to perform global average pooling on the feature layer of the input module, (2) to add two fully connected neural networks and conduct normalization.

#### Feature Extraction Network

Feature extraction is an important part of target detection. The number of target detection frames, classification accuracy, and the detection efficiency is directly affected by the feature extraction network. The backbone network of the YOLOv4 is CSPDarkNet53, which is composed of the resblock body module, one-time down-sampling, and multiple stacking of residual structures.

Initially, input images enter ResBlock by a 3 × 3 convolution channel. Then, the feature map undergoes multiple down-sampling, which is divided into two 1 × 1 convolution layers with stride 1 and enters the partial transition and residual block, respectively. After splicing, the feature map finally passed through convolution to reduce the complexity of calculation and improve the calculation speed.

### Other Compared Models

To compare the detection performance of different CNN models, we trained another three state-of-the-art target detection models YOLOv3, YOLOv5, and Faster R-CNN.

YOLO model was first proposed by Redmon in 2016. YOLOv3 is the third iterating version and has a great improvement on the detection accuracy and speed (Redmon and Farhadi, 2018). YOLOv5 introduced multi-scale network detection to furtherly enhance the model flexibility (Glenn, 2020). Faster R-CNN is a two-stage target detection model, which combines the candidate region generation stage with the classification stage, and can achieve a high detection accuracy (Girshick, 2015).

### Model Training

All models run on a PC with an Intel Core i7-9800xCPU @ 3.8 GHz and 3 GeForce GTX 1080Ti. The operating system is Linux16.04. YOLO series model and Faster R-CNN model run on tensorflow framework.

### Evaluation Metrics

To evaluate the detection effect of the YOLO-MPNet model, we use precision (*P*), recall (*R*), and *F*_1_ as evaluation indicators. Precision indicates the proportion of the target pests that are correctly detected among all detected targets. Recall indicates the proportion of correctly detected pests among the target pests. *F*_1_ is a comprehensive evaluation index of precision and recall, which is used to evaluate model performance when precision and recall are in conflict. The higher the *F*_1_ value, the better the balance of precision and recall. The formulas are as follows.


(1) $$P=\frac{\mathrm{number}\;\;\mathrm{of}\;\;\mathrm{correctly}\;\;\mathrm{detected}\;\;\mathrm{pests}}{\mathrm{total}\;\;\mathrm{number}\;\;\mathrm{of}\;\;\mathrm{detected}\;\;\mathrm{targets}}$$

(2) $$R=\frac{\mathrm{number}\;\;\mathrm{of}\;\;\mathrm{correctly}\;\;\mathrm{detected}\;\;\mathrm{pests}}{\mathrm{total}\;\;\mathrm{number}\;\;\mathrm{of}\;\;\mathrm{target}\;\;\mathrm{pests}}$$

(3) $$F_{1}=2\times \frac{P\times R}{P+R}$$

In our work, the pest detection speed is very important in pest occurring peaks. To evaluate the detection speed of different models, frames per second (FPS) is calculated.

## Results

### Detection Results of Different Models

Table 2 presents the detection results of three migratory pests on the same test set using YOlOv3, YOLOv4, Yolov5, Faster R-CNN, YOLOv4 with OSW and YOLO-MPNet with OSW.

**Table 2:** The detection results of different models for migratory pests.

Detection models	Precision (%)			Recall (%)			*F*_1_ (%)			FPS
	*C. medinalis*	*S. furcifera*	*N. lugens*	*C. medinalis*	*S. furcifera*	*N. lugens*	*C. medinalis*	*S. furcifera*	*N. lugens*	
YOLOv3	70.13	60.69	64.51	57.36	45.13	48.65	63.10	51.76	55.47	0.89
YOLOv4	73.66	66.72	71.27	60.13	55.24	59.24	66.21	60.44	64.70	0.95
YOLOv5	71.26	63.54	69.22	59.21	52.51	56.32	64.68	57.50	62.11	1.02
Faster R-CNN	72.21	54.61	55.76	59.68	47.23	48.11	65.35	50.65	51.65	0.32
YOLOv4 with OSW	81.58	76.42	79.46	82.39	74.63	77.66	81.98	75.51	78.55	0.68
YOLO-MPNet with OSW	94.14	85.82	88.79	91.99	82.47	85.00	93.05	84.11	86.85	0.66

YOLOv4 achieves the higher precision rate, recall rate, and *F*_1_ of three pests than YOLOv3, YOLOv5 and Faster R-CNN. The precision rate of *C. medinalisare*, *S. furcifera*, and *N. lugens.* are 73.66, 66.72, and 71.27%, respectively, and their recall rate are 60.13, 55.24, and 59.24%, respectively. In our mind, the two-stage model Faster R-CNN should have higher precision than one-stage model YOLOv4. As it can be seen, Faster R-CNN seems to be an unsatisfied approach in our pest detection task. Although the FPS of YOLOv5 is higher than YOLOv4, we consider both the precision rate and FPS. So we select the YOLOv4 as an original model which is improved.

The YOLOv4 with overlapping sliding window method effectively improves the precision and recall rates of three pests. The precision rates of *C. medinalisare*, *S. furcifera*, and *N. lugens* are increased by 7.92, 9.7, and 8.19% respectively, their recall rate are increased by 22.26, 19.39, and 18.42%, respectively. Because the sliding window processing method during image preprocessing increases the area ratio of each target pest in the subimages, which helps to extract more abundant features of small target pests and reduce the missing detection.

The searchlight trap attracts a large number of non-target insects. Some insects are similar to target pests, which results in false detection. The improved model YOLO-MPNet with a SENet attention mechanism achieves better detection effects of three migratory pests than YOLOv4 after the same sliding window method is processed on original images. The precision rates of three pests of *C. medinalisare*, *S. furcifera*, and *N. lugens* are increased by 12.56, 9.4, and 9.33% respectively, their recall rates are increased by 9.6, 7.84, and 7.34%, respectively. It proves that the SENet channel attention mechanism can effectively decrease false detection between target pests and interference pests.

### Precision-Recall Analysis

**Figure 5:**
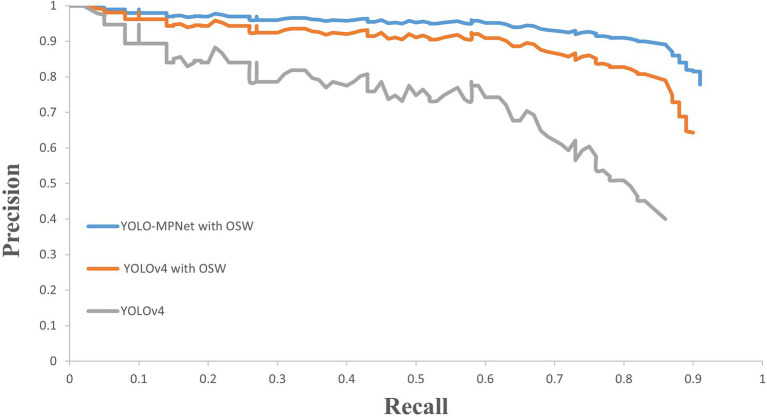
PR curves for different models.

To investigate the false detections and missing detections, PR curves of YOLOv4, YOLOv4 with overlapping sliding window and our improved model YOLO-MPNet with overlapping sliding window are shown in Figure 5. When pest images are processed with overlapping sliding window, the precisions of YOLOv4 and YOLO-MPNet can keep a high value in a big range of recall. So the overlapping sliding window method can effectively reduce pest false detections and missing detections. In general, YOLO-MPNet performs the best on three migratory pest detection with a high precision and recall at same time.

### Visualization of Detection Results

**Figure 6:**
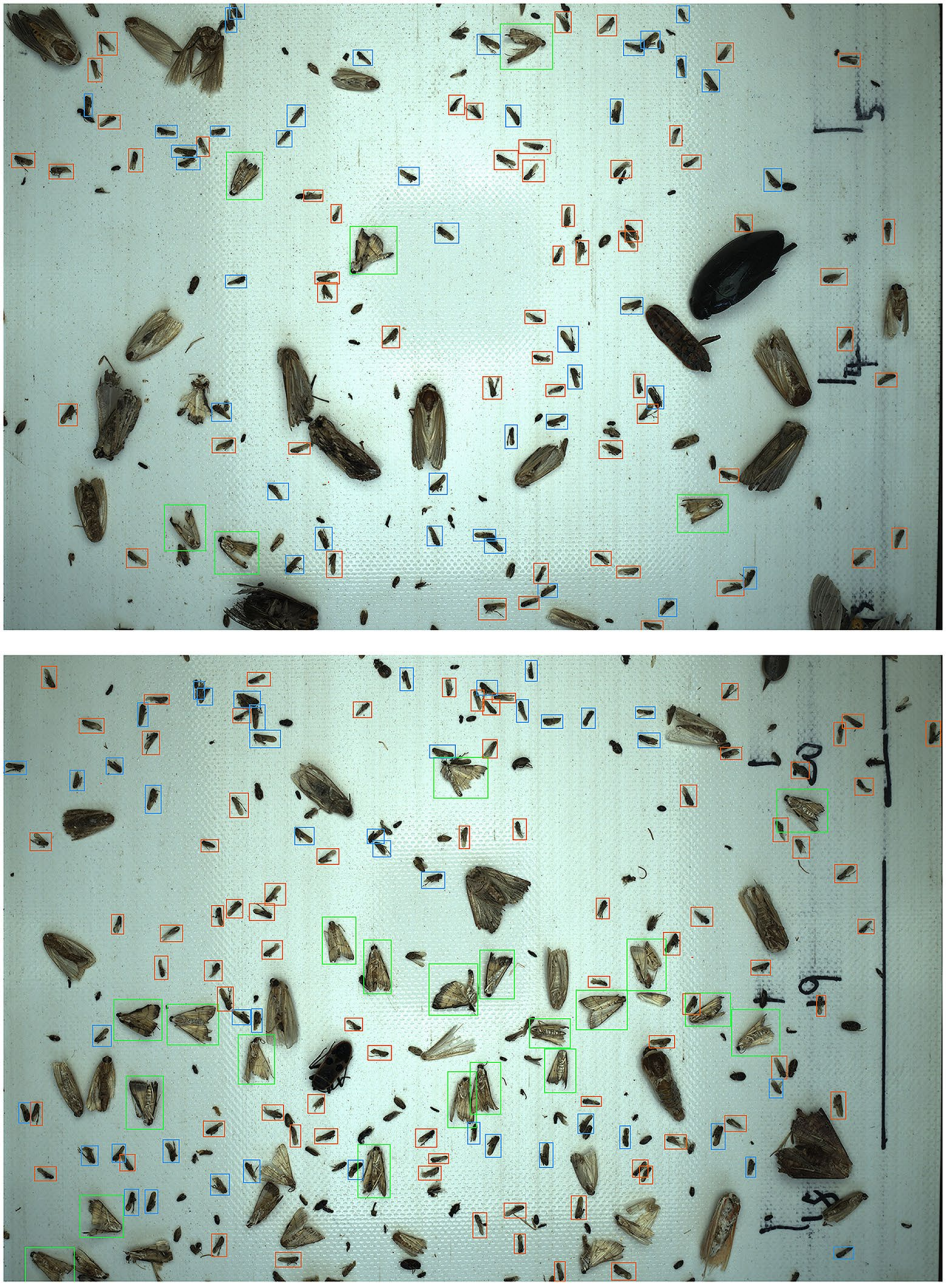
Examples of detected pests. The green, orange, and blue boxes contain *C. medinalis*, *S. furcifera*, and *N. lugens*, respectively.

The detection results of migratory pests are visualized in Figure 6. YOLO-MPNet could detect the three migratory pests well under different insect densities. As it can be seen, the trapped pests could effectively be dispersed by our multilayer insect conveyor belts. Some of occluded pests could be correctly detected by our model.

### Web Client Interface of System

**Figure 7:**
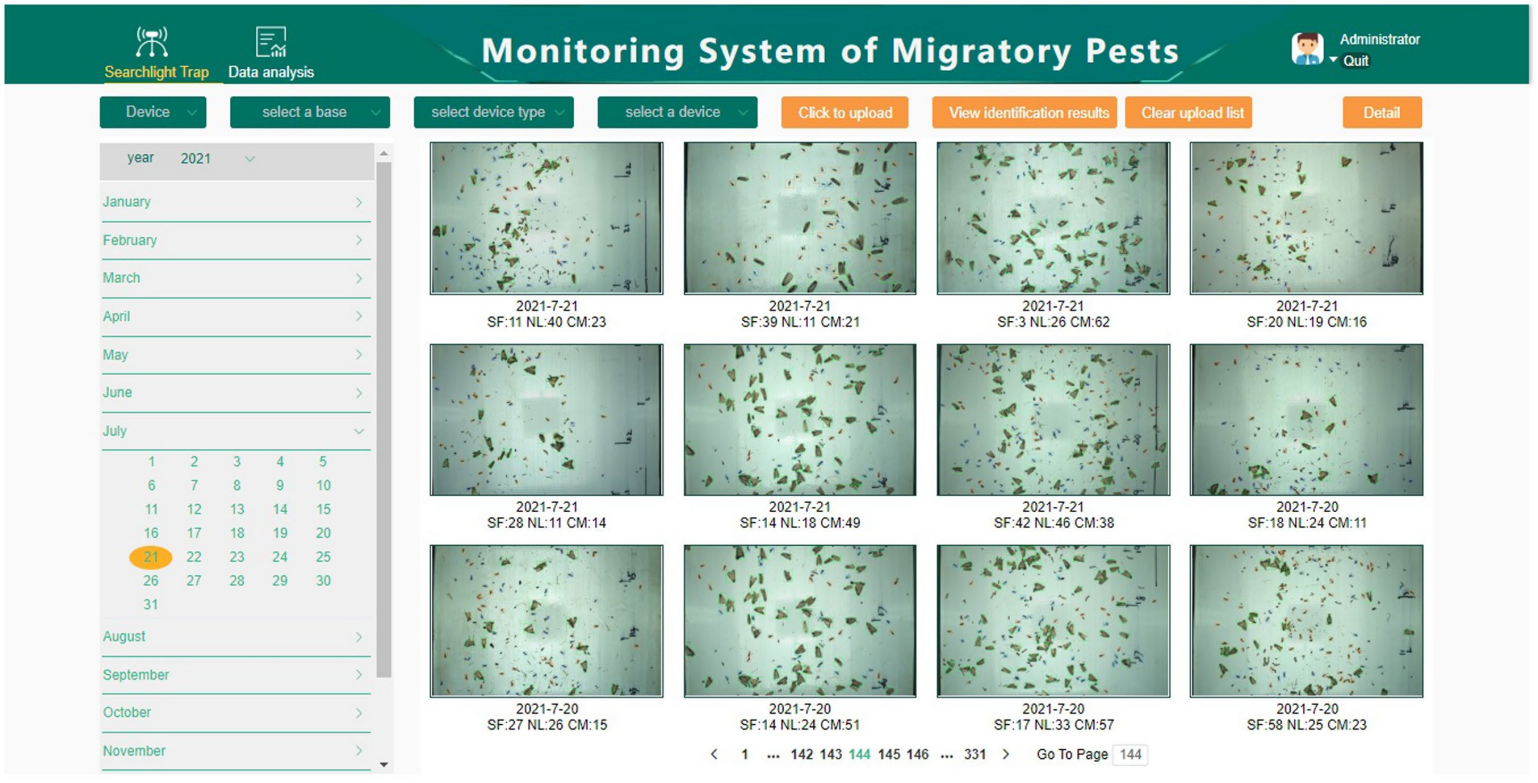
Web client interface of intelligent monitoring system of migratory pests.

The web client interface of the intelligent monitoring system of migratory pests mainly includes user login, automatic identification of migratory pests, equipment management, user management, and data curves. Users can view the detection result images through the web interface and historical monitoring data. Figure 7 shows the web interface of the system and the detected images.

## Conclusion

To realize automatic and accurate identification of rice migratory pests from searchlight traps, we develop an intelligent monitoring system of migratory pests, which is composed of a searchlight trap based on machine vision, an automatic identification model of migratory pests, a Web client, and a cloud server. To identify and count three rice migratory pests (*C. medinalis, S. furcifera*, and *N. lugens*) from a large number of non-target insects trapped by searchlight traps, we propose an improved model, YOLO-MPNet. To solve the problem that the backbone network cannot effectively extract features of small target pests, this paper introduces the overlapping sliding window processing method, which can improve the area proportion of small targets in images and optimize the identification effect of small target pests. At the same time, the feature extraction network is improved by adding the SENet channel attention mechanism. The model’s adaptability to complex backgrounds is strengthened. YOLO-MPNet has achieved higher precision, recall and *F*_1_ values among three species of migratory pests (*C. medinalis*, *S. furcifera*, and *N. lugens*) than the YOLOv3, YOLOv4, YOLOv5, and Faster R-CNN models.

In this paper, only three species of rice migratory pests are identified by our model. In fact, some non-migratory pests are trapped by the searchlight traps. In future work, more species of pests from searchlight traps will be studied.

## Data Availability Statement

The raw data supporting the conclusions of this article will be made available by the authors, without undue reservation.

## Author Contributions

GS, HL, and QY proposed the detection model. GS, HL, SL, QY, and JX wrote and revised the manuscript. SL, BY, JL, and JT contributed to paddy fields and manual data annotation. GS, HL, and ZF developed the system software. All authors contributed to the article and approved the submitted version.

## Funding

This study is supported by the National Key Research Program of China during the 14th Five-Year Plan Period (no. 2021YFD1401100) and the Natural Science Foundation of Zhejiang, China (no. LY20C140008).

## Conflict of Interest

The authors declare that the research was conducted in the absence of any commercial or financial relationships that could be construed as a potential conflict of interest.

## Publisher’s Note

All claims expressed in this article are solely those of the authors and do not necessarily represent those of their affiliated organizations, or those of the publisher, the editors and the reviewers. Any product that may be evaluated in this article, or claim that may be made by its manufacturer, is not guaranteed or endorsed by the publisher.
